# Supplementary material for: Using a Simple Magnetic Adsorbent for the Preconcentration and Determination of Ga(III) and In(III) by Electrothermal Atomic Absorption Spectrometry
Source: Molecules. 2023 Mar 10;28(6):2549. doi: 10.3390/molecules28062549 (PMC10052651; doi:10.3390/molecules28062549)
Supplement: Supplementary file 1 [file molecules-28-02549-s001.zip › molecules-2266245-supplementary.pdf]

Using a simple magnetic adsorbent for the preconcentration and determination of Ga(III) and In(III) by electrothermal atomic absorption spectrometry

Yesica Vicente-Martínez, María José Muñoz-Sandoval, Manuel Hernández-Córdoba and Ignacio López-García

Department of Analytical Chemistry, Faculty of Chemistry, Regional Campus of International Excellence "Campus Mare Nostrum", University of Murcia, 30100-Murcia, Spain

Supplementary material

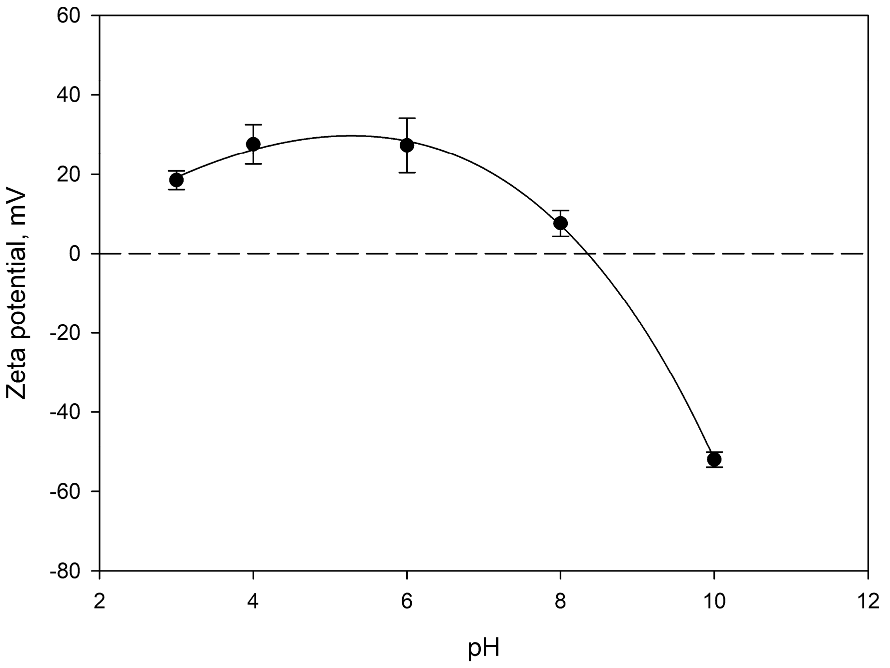

Figure S1. Zeta potential of ferrite particles synthetized as indicated in Experimental measured with dynamic light scattering (DLS).

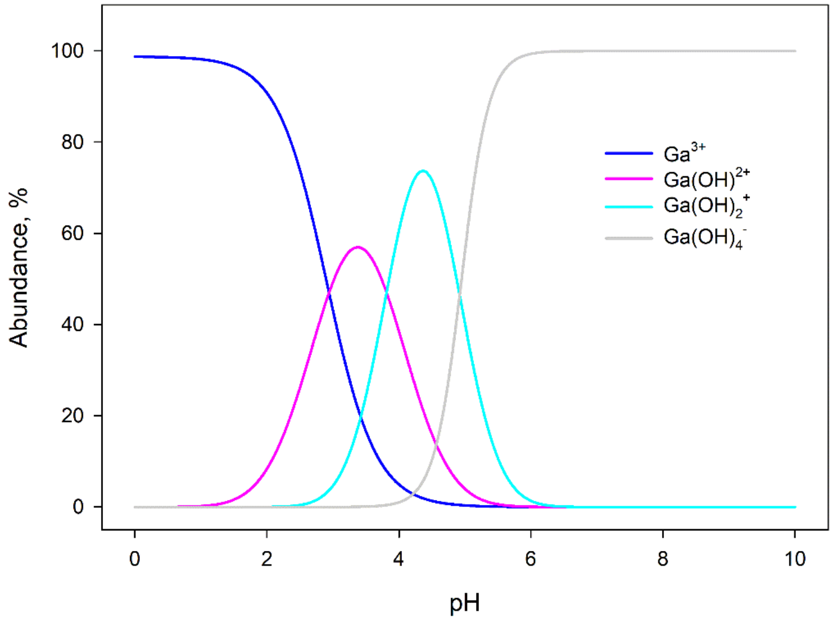

Figure S2: Distribution diagram of the hydrolysed forms of Ga(III) considering the polynuclear species, adapted from [1].

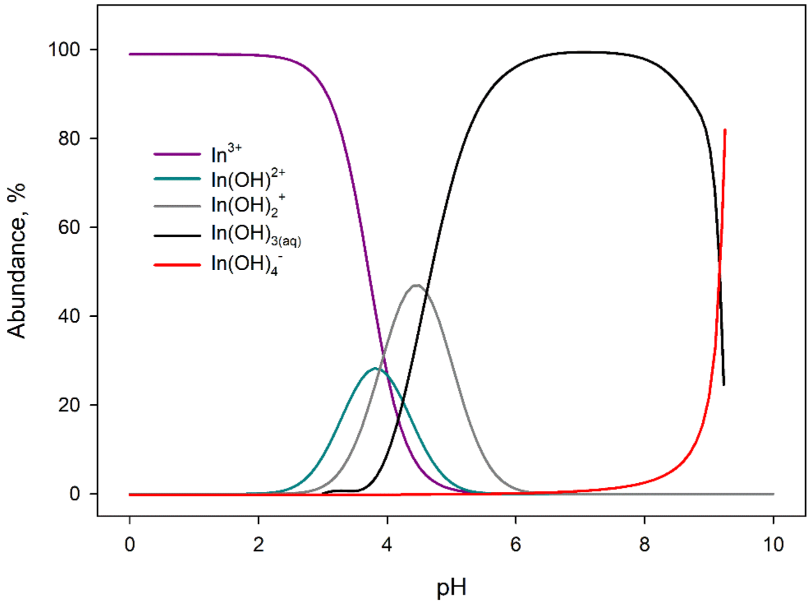

Figure S3: Distribution diagram for hydrolysed forms of In(III), adapted from [2].

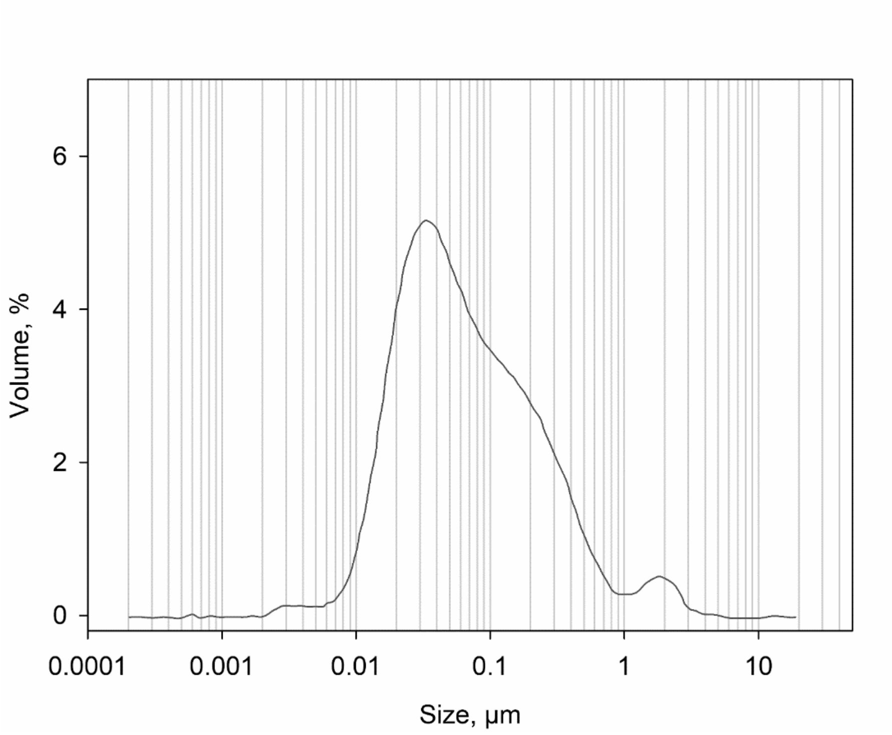

Figure S4. Dynamic Light Scattering (DSL) particle size distribution analysis obtained from ferrite particles synthetized as indicated in the work.

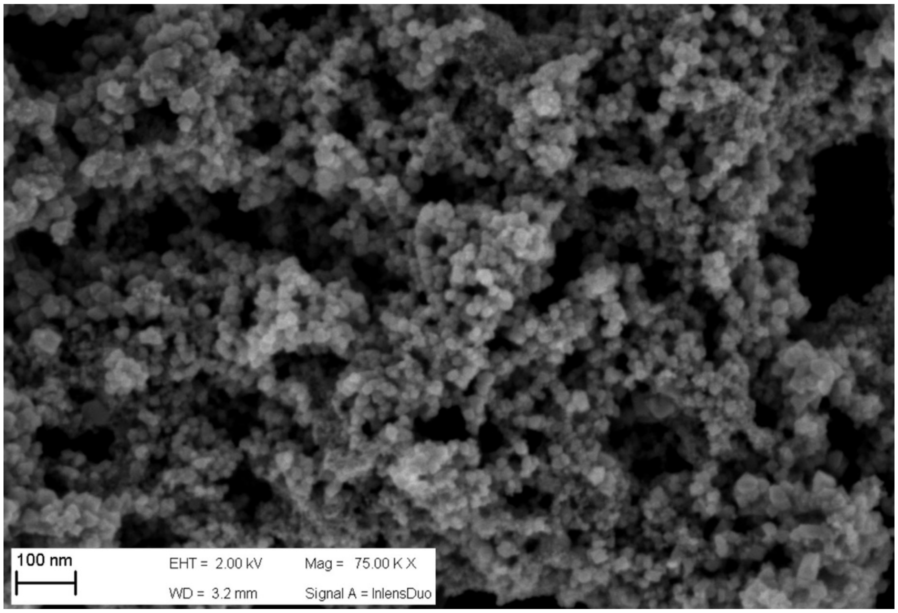

Figure S5. FESEM image for Fe_3O_4 .

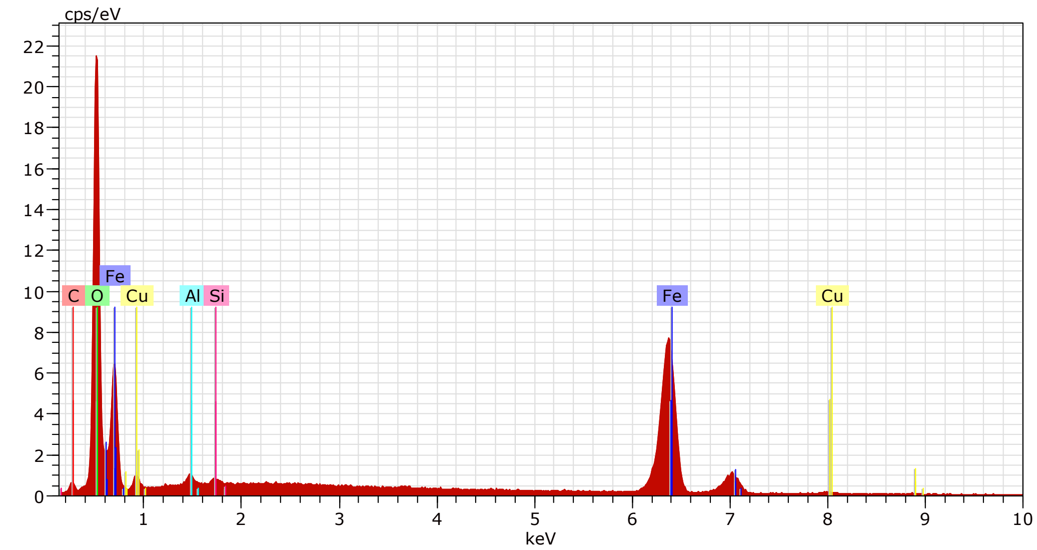

El	AN	Series	unn. C [wt.%]	norm. C [wt.%]	Atom. C [at.%]	Error (1 Sigma) [wt.%]
C	6	K-series	1.73	1.88	4.63	0.39
O	8	K-series	29.98	32.73	60.43	3.47
Al	13	K-series	0.55	0.60	0.65	0.06
Si	14	K-series	0.26	0.28	0.29	0.04
Fe	26	K-series	57.32	62.58	33.10	1.72
Cu	29	K-series	1.77	1.93	0.90	0.11
		Total:	91.59	100.00	100.00	

Figure S6. EDX spectra and atomic concentration tables corresponding to the area shows in Figure S5, for Fe_3O_4.

Reference

1. Hacht, B. Gallium(III) ion hydrolysis under physiological conditions. *Bull. Korean Chem. Soc.* 2008, 29, 372–376.
2. Wood, S.A.; Samson, I.M. The aqueous geochemistry of gallium, germanium, indium and scandium, *Ore Geol. Rev.* **2006**, *28*, 57–102. https://doi.org/10.1016/j.oregeorev.2003.06.002.
